# 294 Comparison of Healthcare-Associated Infection Rates Using Disposable and Reusable Isolation Gowns

**DOI:** 10.1017/ash.2026.10652

**Published:** 2026-06-23

**Authors:** Victoria Gavaghan, Robert Citronberg, Jill Argotsinger

**Affiliations:** 1 Advocate Health; 2 Advocate Lutheran General Hospital

## Abstract

**Background:** Carbapenem-resistant Acinetobacter baumannii (CRAB) infections present treatment challenges due to host-related factors, infection source, and complex resistance mechanisms. Attribution of poor clinical outcomes to suboptimal therapy versus underlying host factors remains unclear. Evidence supporting an optimal treatment strategy is limited. Current Infectious Diseases Society of America (IDSA) guidance recommends high-dose ampicillin-sulbactam in combination with an additional agent, including minocycline, as a treatment option. In 2025, the Clinical & Laboratory Standards Institute (CLSI) lowered the A. baumannii minocycline susceptibility breakpoint from ?4 to ?1 mcg/mL. Despite this change, our health system’s standard practice includes high-dose ampicillin-sulbactam plus minocycline for invasive CRAB infections. Given the updated CLSI breakpoint, this study evaluated clinical outcomes among patients with CRAB infections treated with minocycline. **Methods:** This was a retrospective cohort study of hospitalized adult patients with CRAB isolates who received minocycline within a large integrated health system from June 1, 2024, to June 1, 2025. Patients were excluded if isolates were deemed colonization by provider documentation or cultures were obtained in the outpatient setting. The primary outcome was clinical failure defined as need for antibiotic escalation, lack of microbiologic cure, 90-day readmission requiring retreatment, or 90-day all-cause mortality. **Result:** We identified 137 CRAB isolates from 128 patients; majority were male (84, 61.3%) with a mean age of 63 ± 14.5 years. ICU admission occurred in 57 patients (39.4%) and the mean Charlson Comorbidity Index was 6.1 ± 2.5. The most common infection sources were respiratory (75, 54.7%), skin and soft tissue/osteomyelitis (34, 24.8%), and bloodstream (19, 13.9%). Most infections were polymicrobial (95, 69.3%). Combination therapy was used in 102 cases (74.5%). Based on updated CLSI breakpoints, only 35% of isolates were minocycline susceptible versus 84% susceptibility to previous breakpoints. Clinical failure was similar regardless of minocycline susceptibility (52.1% with MIC ?1 mcg/mL vs 51.7% with MIC <1 mcg/mL, p=0.964). In contrast, clinical failure was more frequent among patients with CRAB bacteremia (14/19, 73.7%; p=0.04), 18 of which had isolates with a minocycline MIC **Conclusion:** Following the 2025 CLSI breakpoint revision, most CRAB isolates were identified as minocycline resistant. Clinical outcomes did not differ between groups based on minocycline susceptibility. Outcomes were poor however for sterile-site infections despite documented in-vitro susceptibility. These findings highlight the need for careful differentiation between colonization and true infection at non-sterile sites and emphasize the limitation of MIC-based susceptibility in predicting clinical outcomes of CRAB infections.

